# Beyond Labelling: What Strategies Do Nut Allergic Individuals Employ to Make Food Choices? A Qualitative Study

**DOI:** 10.1371/journal.pone.0055293

**Published:** 2013-01-29

**Authors:** Julie Barnett, Konstantina Vasileiou, M. Hazel Gowland, Monique M. Raats, Jane S. Lucas

**Affiliations:** 1 Department of Information Systems and Computing, Brunel University, Middlesex, United Kingdom; 2 Allergy Action, Farnborough, United Kingdom; 3 School of Psychology, Faculty of Arts and Human Sciences, University of Surrey, Guildford, Surrey, United Kingdom; 4 Clinical and Experimental Sciences, Faculty of Medicine, University of Southampton, University Hospital Southampton NHS Foundation Trust, Southampton, United Kingdom; Universidad de Granada, Spain

## Abstract

**Objective:**

Food labelling is an important tool that assists people with peanut and tree nut allergies to avoid allergens. Nonetheless, other strategies are also developed and used in food choice decision making. In this paper, we examined the strategies that nut allergic individuals deploy to make safe food choices in addition to a reliance on food labelling.

**Methods:**

Three qualitative methods: an accompanied shop, in-depth semi-structured interviews, and the product choice reasoning task – were used with 32 patients that had a clinical history of reactions to peanuts and/or tree nuts consistent with IgE-mediated food allergy. Thematic analysis was applied to the transcribed data.

**Results:**

Three main strategies were identified that informed the risk assessments and food choice practices of nut allergic individuals. These pertained to: (1) qualities of product such as the product category or the country of origin, (2) past experience of consuming a food product, and (3) sensory appreciation of risk. Risk reasoning and risk management behaviours were often contingent on the context and other physiological and socio-psychological needs which often competed with risk considerations.

**Conclusions:**

Understanding and taking into account the complexity of strategies and the influences of contextual factors will allow healthcare practitioners, allergy nutritionists, and caregivers to advise and educate patients more effectively in choosing foods safely. Governmental bodies and policy makers could also benefit from an understanding of these food choice strategies when risk management policies are designed and developed.

## Introduction

Food allergy is an important public health problem [1]. Although there are difficulties in estimating prevalence, and there is some variation between countries [2], in Europe and the United States food allergies are estimated to affect up to approximately 8% of children and 2% of adults [3], [4]. Peanut and tree nuts are the most common cause of severe and life-threatening food allergic reactions [5]. The prevalence of peanut allergy, tree nut allergy or both (henceforth ‘nut allergies’) is around 1% in North American and UK populations [6], [7], [8], [9] and seems to be increasing [7], [8], [10], [11]. Between 1999 and 2006, 18 out of 48 deaths which were caused by allergic reactions to foods in the UK [12], and more than 90% of fatal reactions in the US between 1994 and 1999, were provoked by peanuts or tree nuts [13]. For ease – and despite the fact that peanuts are actually legumes – the word ‘nuts’ is henceforth used as a convenient generic.

The quality of life of children and adolescents with a nut allergy including their families can be severely compromised [14], [15] since constant vigilance about diet needs to be exercised, whilst the risk of accidental exposure and the fear of a fatal reaction cannot completely be eliminated and controlled [16]. Parents of children with a nut allergy report higher levels of impairment in their quality of life and more disruptions of familial and social relations than do parents of children with a rheumatologic disease [17]. Similarly, higher levels of disease-related anxiety were found in children with a nut allergy compared with children with diabetes [18]. Recent research in adults has revealed the difficulties and challenges that nut allergic individuals face when eating out [19] and when travelling abroad [20] where a series of often competing considerations need to be balanced.

The clinical manifestation of allergic reactions includes symptoms affecting the skin (e.g. urticarial), respiratory tract (e.g. dyspnoea, throat tightness), gastrointestinal tract (e.g. vomiting, diarrhoea), and/or the cardiovascular system (e.g. hypotensive shock) [3]. Anaphylactic reactions are life-threatening episodes involving the cardiovascular or respiratory systems, which should be treated immediately by injection of intramuscular adrenaline [3], [4]. Nut allergies are not currently curable and consequently management primarily involves effective allergen avoidance and the provision of self-injectable adrenaline for severe reactions [1], [21]. However, patients do not always carry their adrenaline devices or use them appropriately [22], [23], and even when correctly administered, reactions can be fatal. Therefore, recognizing and avoiding foods containing nuts remains the key self-management strategy.

Labelling on the packaging of food products is an important tool that helps nut allergic individuals to make safe purchases. Although consumers do not normally pay much regard to labels, specific dietary requirements, such as food allergies, increase their attention to food labels [24]. Nonetheless, nut allergic people often encounter a series of difficulties in identifying correctly the allergens on the labels [25], [26], and in interpreting advisory labelling such as ‘may contain’ [27]. Moreover, an examination of 20,241 products in US revealed the ambiguities and the variety of terminologies used in advisory labelling [28] confirming the challenges that nut allergic individuals face when buying foods. Recently, qualitative research in the UK [29], [30] examined the ways in which nut allergic adults used information on the packaging to inform their food choices, corroborating many of the difficulties mentioned previously in the literature. It is evident, however, that labelling was used in conjunction with a series of other decision making strategies. But, whilst food choice strategies and ‘rules of thumb’ have been studied extensively among consumers in general [31], [32], [33], [34], little is known in relation to nut allergic individuals. A recent focus-group study compared the food choice behaviours and eating experiences of adults diagnosed with food allergies to those of non-allergic consumers. The results demonstrated that food allergic people usually encounter difficulties in finding safe foods; their experiences around eating are less satisfactory and spontaneous since a high level of organisation and preparation is constantly required [35].

This paper has sought to characterise the nature of strategies employed by nut allergic individuals when choosing foods that extend beyond labelling. By using a variety of qualitative methods, we aimed to examine in depth how nut allergic individuals reason about food risk and how they choose and purchase foods.

## Methods

### Ethics Statement

The research received ethical approval from the National Research Ethics Service (09/H1109/64) and the University of Surrey Ethics Committee.

### Study Population

The study population included individuals, aged 16 or over, with a clinical history of reactions to peanuts and/or tree nuts which was compatible with IgE-mediated allergy. The participants were recruited from three sources in South England: (a) the paediatric and adult allergy clinics at University Hospital Southampton NHS Foundation Trust, (b) three General Practitioners (GP) Surgeries, and (c) the staff and students at the University of Surrey. Letters were sent to 411 individuals explaining the aim of the research and inviting them to complete a screening questionnaire. For the interested reader, the screening questionnaire is provided as File S1. Seventy-seven respondents returned this (response rate 18.73%) of which 54 were eligible to take part following examination of the information by an allergy specialist (JSL). Of the 54 eligible individuals, 32 consented to participate in the study. Participants' consent was obtained in written form.

Participants' eligibility, based on the information provided in the postal screening questionnaire, was assessed by the allergy specialist as follows: respondents were required to be aged 16 years or older with a clinical history compatible with IgE-mediated reactions to peanuts or tree nuts. Participants recruited from the specialist allergy clinic had positive skin prick tests and/or specific IgE measurements. Recruits from the University of Surrey and from primary care settings were required to have been diagnosed with peanut or tree nut allergy by a medical practitioner, and prescribed rescue medication. Individuals with allergies or intolerance to foods in addition to peanut or tree nuts were excluded because this would affect their consumer choices. The exception was inclusion of participants with oral allergy syndrome (OAS) to fruits and/or vegetables. Unlike egg or milk for example, avoidance of fruit and vegetables was unlikely to create significant dilemmas during the shopping tasks.

Of the 32 participants who took part in the study, 9 were males and 23 females, aged between 16 and 70 years old. Five participants had only peanut allergy, 9 had only tree nut allergy, and 18 had both. The severity of their worst-ever reaction was rated as mild, moderate or severe using a classification previously employed for peanut allergy [36]. Eighteen participants described severe reactions, 12 moderate and 2 mild.

### Methods of Data Collection

Three different qualitative methods were used to collect data:

#### A. The accompanied shop (AS)

Participants were followed, observed and audio-recorded in their routine weekly shop by an experienced qualitative researcher. Accompanying consumers while shopping is an established technique in consumer studies that enables direct observation of actual behaviour [37]. Participants in this research were additionally asked to think aloud about their purchase decisions, having being familiarised with and trained in the ‘think aloud’ method. File S2 provides the details of how participants were trained to the ‘think aloud’ protocol and how they were instructed to conduct the accompanied shop. To ensure that the accompanied shop was as naturalistic as possible, the researcher's input at this stage was minimal, consisting mainly of prompts such as ‘what are you thinking?’ when participants stopped thinking aloud.

The ‘think aloud’ technique allows the elicitation of a concurrent with-the-behaviour-observed verbal report. Ericsson & Simon's [38] classical work advocated the value of verbal data deriving from introspection for the examination of the cognitive processes involved in problem-solving scenarios. Since then, the ‘think aloud’ technique has been used extensively in several fields, including food choice research [39] usually after being adapted to serve particular research questions and in combination with other techniques [40].

#### B. A semi-structured interview (SSI)

Issues arising during the accompanied shop were followed up in a face-to-face interview. This allowed us to examine interesting behaviours noted by the researcher during the accompanied shop that was relevant to our research questions (e.g. avoiding certain products or even parts of the supermarket). The SSI also explored the ways the participants were managing their food choices and consumption across various eating-related situations (e.g. eating-out, holidays), as well as the history of their allergies and their views on labelling.

#### C. The Product Choice Reasoning Task (PCRT)

Participants were presented with 13 products, each of which embodied a recognized dilemma for people avoiding nuts/peanuts. These are described in detail in Barnett et al [29]. Participants were asked whether and why they would or would not consume each one of the products.

Fuller detail of the methods can be found in Barnett et al [41].

Employing this configuration of methods allowed the in-depth examination of both reported and actual food choices and the reasoning behind them. All research phases were audio recorded and fully transcribed. The accompanied shop was directly followed by the interview and the Product Choice Reasoning Task which were conducted in participants' homes.

### Analytic Procedure

Thematic analysis [42], [43] was used to explore the data. Given that the aim was to examine what strategies the nut allergic individuals are employing to make sense of, and manage the risk of allergic reactions when choosing food, a realist epistemological stance to participants' speeches was adopted. This means that people's accounts were considered as being reflective of their thoughts, cognitions, and reported behaviours and that language provided the means through which the researchers were able to access these.

Initially, the researchers (JB and KV) familiarised themselves with the data through repeated reading of the transcripts, and noted interesting and relevant-to-the-research-question points. Further analysis was conducted during a series of regular meetings. Codes were formed and assigned, assisted by computer software [44], to the relevant textual segments. Finally, themes and subthemes were developed by aggregating semantically identical codes; further revision and refinement of the themes took place in order to be internally homogenous, distinct from each other [45] and reflective of the data.

## Results

Risk reasoning and food choice practices were located in relation to three main themes: (a) *product qualities*, (b) *past experience*, and (c) *sensory appreciation*. Food choices, however, were not exclusively defined by safety considerations; participants reported other considerations, which often competed with those pertaining to risk forming a fourth theme, named *beyond safety*.

Respondents' quotes below are identified by a. their coding number (e.g. 4015), b. their sex (M for males, F for females), c. the severity of allergy (S for severe, M for moderate and MI for mild), d. their age, and e. the corpus of data from which the quote is drawn (Accompanied shop – AS, Semi-Structured Interview – SSI, and Product Choice Reasoning Task - PCRT).

### Product Qualities

Several product-related indicators were employed, sometimes in combination, to inform participants' risk assessments. These concerned the *product category*, the *brand*, the *producer and/or the provider* of the food, and the *country of origin*, and operated to provide guidance as to the desirability of purchase and consumption.

The broader *product category* was among the most salient categorisations that informed risk judgments. Certain product categories, such as bakery, chocolate, cereals, desserts, processed or ready-made meals, were considered as particularly problematic, as they were strongly associated with nuts, signalling the necessity for closer examination usually by looking the labels. Hesitation and worry around consumption of these products was often reported, while total rejection was not uncommon:

*If it's like a savoury type thing, particularly bready stuff, for some reason, that's what freaks me out a little bit, and I'll just say…bready things, biscuits, cakes, I'll just say, “No thanks”. (4015, MS34, PCRT)*

Fresh vegetables, fruits, and dairy, on the other hand, were product categories that rarely posed any safety concerns and were generally trusted immediately as safe.

The *brand* also informed risk reasoning. Well-known and reputable brand names clearly functioned to indicate safety. Notably, in some instances a trusted brand acted as such a strong indicator of safety that it could override precautionary labelling or the influence of a problematic product category:

*Yeah, I'd eat these. Normally – oh, it says “Not suitable for peanut allergy sufferers”… I'd probably still eat them though because if they're – like [brand name] is a trusted name, and I highly doubt they actually would. I'd probably still eat them, yeah. (1011, FS18, PCRT)*

Conversely, products associated with a lesser known brand, even when they were not problematic *per se*, raised suspicions which were resolved either through avoidance or further checks of labels:

*For a first look, I'd probably be like, “Oh yeah, I can eat them,” and then I'd just be like, “Oh, hang on, it's not a main brand – I probably should have a look at it.” (1008, FM16, PCRT)*

The trust accorded to well-known brands was associated with the assumed safety standards of the manufacturing processes and the perceived thoroughness and adequacy of labelling practices, although participants did not claim any knowledge of manufacturing or labelling processes:

*That's a brand that I would be comfortable that their food labelling and their standards are adequate. (1029, FS26, PCRT)*

The *provider* of the products – which in some cases coincided with the producer – was an additional resource for risk judgments. Big companies such as supermarkets were often trusted due to the belief that they have strong interests to protect their reputation and enough resources to check the products:

*…so if I'm buying any sort of products where it relates to nuts, I would definitely only buy them in the supermarket, because I'm pretty much trusting that someone is checking and that the risk of me finding them in the food is less. (3008, FM45, SSI)*

A smaller number of participants, however, perceived small, and usually local, producers or providers as more trustworthy than big companies.

The last product quality repeatedly articulated in risk reasoning was the *country of origin*. Foreign products were almost always treated with hesitation and were avoided compared to native products:

*Em, anything that's foreign is a no – I just won't buy it. (4015, MS34, SSI)*

Even if the particular allergen was apparently not an ingredient the overarching designation as foreign led to rejection. Foreign products activated beliefs that in other countries there is a lower level of awareness or recognition of the problem of nut allergies and a higher tendency to use nuts in products:

*They look like they're probably made somewhere else because the initial language here is not English. I have no idea how they…if they have any concept of allergies. (5009, FS38, PCRT)*

Reluctance to consume foreign products was also explained in terms of unfamiliarity with the standards of production and regulation applied in other countries:

*To be honest, if it was a foreign product, which had equally good labelling, I might be more reluctant because I'm not familiar with the standards that they would be obliged to comply with (1029, FS26, PCRT).*

### Past Experience

Past experience was a second, and particularly powerful, resource for nut allergic individuals that dictated safe food choices in an efficient and confident manner. Experiential knowledge accumulated over the course of years invited absolute trust in foods, often without reference to labels:

*I could almost say yes without looking at the ingredients for these ones because I've eaten them lots of times before. (1068, FM34, PCRT)*

Several participants were alert to the possibility that manufacturers may change product ingredients. Some reported that they still checked the labels for potential changes, even though past experience warranted safe consumption, but others ignored this possibility, especially when they had recently consumed the product:

*If I know that it's something I've had quite recently, then I'm not bothered about it. I'll just have it straightaway, pick it up without reading it. (1016, FS18, SSI)*

Importantly, uneventful past experience of consuming a product was such a powerful indicator of future safe consumption that precautionary labelling was ignored or discounted:

*Yeah, I can see here it says that it's not suitable for peanut allergy sufferers and it may have traces of peanut or other nuts, but I've never ever had an allergy to [product name], and so my automatic reaction is yeah, I'd try it. (3008, FM45, PCRT)*

Conversely, novel products led, at least some participants, to attend to ‘contains’ or ‘may contain’ labelling.

Positive experiences of consumption led to the routinisation of food choices and the establishment of habitual food purchase patterns. Although participants recognized that this strategy limited the trial of new foods, they were generally content since time and effort were saved:

*So yes, I do tend to stick with things I know, and probably not adventurous with trying many new things [laughing]! (1042, FS26, SSI)*

Negative past experiences were particularly instructive in creating an often intense aversion and rejection of the associated foods. This was often not simply restricted to the particular product that had caused the allergic reaction but was rather generalised to include other exemplars of the broader category in which the problematic food was classified, for example unrelated products within the same brand or product category, with similar ingredients, or even similar labelling:

*It was a type of chocolate bar from [supermarket name] and I had a reaction to it, even though it just said “may contain”, and then I never ate anything that said “may contain” again. (1112, FS21, SSI)*

### Sensory Appreciation

Reliance on senses such as taste, sight and smell was an additional and trusted strategy the participants employed to assess the riskiness of foods. This strategy was often used when the food was novel and there was thus some uncertainty around its safety. A sensory check provided a rapid initial assessment, yet the resulting evidence was in many cases regarded as unequivocal.

The PCRT was particularly revealing in showing that some products were directly associated with nuts simply from the way they looked. This created strong feelings of aversion to a degree that the product was ultimately rejected without further examination or seeking further validation through labelling:

*It looks too much like nuts, so no. Actually, seeing – just on what it looks like, I wouldn't even, in the real world, read any ingredients. (4015, MS34, PCRT)*

The texture of the product was an additional cue signifying risk. The more plain and smooth the product looked the less risky it was perceived to be. By contrast, a granular texture in the product caused concern and activated avoidance:

*Well, I think it looks risky because there's lots of bits in [laughing]! That sounds simplistic, but over the years, I've learnt that means trouble. (1031, FM30, PCRT)*

Olfactory evaluation was also used for risk judgments and was very much trusted as a signal of danger. Participants smelt the products and some even claimed that they had developed an acute sensitivity to detect nuts or products containing nuts with their odour even ‘from a mile off’. This then provoked again avoidance and rejection of the product:

*I'm just more aware, and I would trust my senses more with regards to my sense of smell, very much so…Those smells immediately send warning bells off in my head. (4015, MS34, SSI)*

Finally, a sort of ‘taste test’ was recurrently reported. Here participants tried a little bit of the product, waited to see whether there were any adverse reactions and depending on the outcome of the trial, they then either consumed or rejected the food. The taste test was deployed when participants felt uncertain about the safety of the food or lacked previous experience. For some, this strategy was still used, even when they knew that the product was safe, thus providing further reassurance:

*Even when I come to eat it, even if I know there's not actually any nuts in it, I'll taste a little bit and then wait a few minutes to see if anything happens…just to be really sure. (4001, FS24, SSI)*

### Beyond Safety

Arguably safety was the primary consideration in food choices. However, other factors influenced decision making. These pertained firstly to other product attributes that were taken into account in food purchases, secondly to contextual factors within which the nut allergic individuals assessed the risk of food consumption, and thirdly to their internal –physiological or psychological – states and the broader life stage. Taking into account these parameters led on occasions to a more risk adverse stance, whilst in other instances greater risk was embraced.

Much of the time participants were considering and valuing additional product attributes such as taste, cost, quality, health, convenience and ethics as well as the needs of other people for whom they were shopping. Although on many occasions these considerations were not related to problematic foods, in some instances there was evidence of how participants sought to incorporate these values alongside their risk assessments. Importantly, in the effort to balancing competing values some admitted that they were taking the risk, mainly when they liked the taste of a product:

*Generally I would do, apart from in the case of the soya nuts [laughing], because I like them so I take the risk! (1042, FS26, SSI)*

Assignations of risk were closely enmeshed with the context within which participants were operating. Two dimensions of the context were evaluated; firstly, respondents were more willing and relaxed to assume greater risk when they were close to medical facilities:

*I mean, if I was in the centre of town in a country where I know the medical facilities are good, then I'm much more relaxed, and I'll risk certain things. If I'm up a mountain in the middle of nowhere, even if it seems safe, I would be more reluctant to eat. (1029, FS26, SSI)*

Secondly, the presence of other people by whom participants and their allergy were known, and who were therefore trusted, was a further reassuring parameter that allowed people to take on greater risks:

*Usually, it's with my parents, and I'm happier eating out at a restaurant with my parents because they know me, they know…like no one knows you as well as your parents do – no one ever would. So I'm happier…like when we've been on holidays and stuff, I'd be happier to risk it when they're there because they know everything. They know my medical history. (1112, FS21, SSI)*

Interestingly, while the presence of others sometimes functioned as reassurance, in many instances it was a source of stress, and more particularly embarrassment, due to participants' belief that interrogating others about the content of food violated important social norms. The feelings of embarrassment and the unwillingness of people to attract attention or to offend others often obstructed the approach to managing risk:

*So I did actually have a bit, even though, really, by my rules I set myself, I wouldn't have eaten that normally, but because it was my birthday cake, I felt a bit guilty! (1161, FM22, SSI)*

It was evident that risk judgments made by participants were contingent upon attending to aspects of their internal state, personal characteristics or life situation. For instance, people mentioned that factors, such as hunger or tiredness, could impede a thorough risk assessment:

*If you're tired, you go round the supermarket, you're really wanting to get home, you do occasionally pick things up and you haven't read it clearly. (4013, FS37, SSI)*

Others related the way they managed their allergy to broader life contexts and significant transitions. A woman (1031, FM30, SSI) mentioned that she used to be much more adventurous with food choices before her baby was born as she had more time to check labels and try new foods. Another participant (1198, FM48, SSI) explained the difficulties of allergy being diagnosed as an adult as well as how a busy life with children militated against giving much attention to her own food choices. Finally, a young woman with severe allergy linked the way she managed her allergy to the general rise and fall of her stress levels:

*If I'm very stressed at work or stressed for whatever reason, I'll become more paranoid about it. It's like something that I focus my energy on or relax away from, depending on everything else that's going on in my life! (1029, FS26, SSI)*

## Discussion

Our results indicate the complexity of risk reasoning and the variety of strategies the nut allergic individuals use to inform their decision making. Participants relied heavily on their previous experience of consuming foods and the relevant knowledge they had developed, their senses as well as their evaluations of different product characteristics, namely the product category, the brand, the provider and the country of origin. These results chime with previous research, conducted with non-allergic consumers, which demonstrates that both past experience and sensory assessments of foods constitute particularly useful rules of thumb in food choices [34]. Applying a system of categorisations and evaluating foods accordingly has also been observed with lay people when they attempt to make sense of various food risks [46], [47]. Additionally, the strategies that people use to make food choices are characterised by trust, dictate confident food consumption, and provide efficiency in decision making [34]; findings which are corroborated by our results. At the same time, this study indicates the complexity of the dilemmas that nut allergic people often face when choosing food, also alluded to in recent research with food allergic consumers [35]. The current study has shed further light on the food choice practices of people with nut allergies and adds to previous research that examines the strategies of non-allergic consumers in this area [31], [32], [33], [34].

One of the strengths of the present research is the variety of qualitative methods used that enabled a detailed insight into the complexity of risk reasoning and food management practices [48]. The accompanied shop allowed us to observe the participants while shopping. This access to their shopping practices seeks to address the challenge of obtaining relevant behavioural data and avoids a sole reliance on self-reported attitudes or behaviour. Additionally, since participants at this stage were simply informed that the focus was on their food management strategies rather than framing this in terms of risk, the accompanied shop proved to be an ideal setting within which other product-based considerations (e.g. taste, cost) were revealed thus further reducing the likelihood of socially desirable behaviours. The interview enabled an in-depth examination of issues emerging during the accompanied shop and provided participants with the opportunity to reflect on their reasoning around their food choices and risk managing behaviour. Finally, the Product Choice Reasoning Task was particularly useful in documenting the exact risk thinking as this was being shaped as well as the, often combined, deployment of the various strategies in decision making.

### Implications of the study

This study has clear implications for healthcare professionals (clinicians, allergists, allergy dieticians) that develop management plans and advise patients as to how to avoid problematic foods, while also maintaining a balanced and healthy diet. On the one hand, it is important to recognize that the strategies provide efficiency and confidence in food decision making, reducing the anxiety and thus they are likely to lead to established behaviour patterns. On the other hand, it is also crucial to acknowledge that the strategies are contingent on contextual factors and are additionally influenced by physiological and socio-psychological needs that sometimes impede thorough and extensive risk assessments and consequently lead to accidental ingestion of the allergens. Understanding and taking into account the day to day strategies that shape nut allergic individuals' food choice practices will enable healthcare professionals to advise their patients more effectively by drawing attention to potential pitfalls and by fostering the use of successful and functional strategies.

Food manufacturers, importers, distributors and retailers also have a responsibility to ensure that the foods they sell are safe for all consumers, including those with food allergies. Policy makers and governmental bodies would also benefit from an understanding of these food choice strategies when risk management policies are developed. It is notable for example that people with nut allergies are most likely to turn to labelling in the context of uncertainty when more habitual strategies do not lead to a confident decision. Under these circumstances it is vital that labelling is available, clear and trustworthy.

## Supporting Information

File S1 Screening questionnaire.
(DOCX)

File S2 Think aloud training & accompanied shop instructions.
(DOCX)
