# Supplementary material for: Beyond Labelling: What Strategies Do Nut Allergic Individuals Employ to Make Food Choices? A Qualitative Study
Source: PLoS One. 2013 Jan 29;8(1):e55293. doi: 10.1371/journal.pone.0055293 (PMC3558473; doi:10.1371/journal.pone.0055293)
Supplement: File S1 — Screening questionnaire. (DOCX) [file pone.0055293.s001.docx]

**Screening questionnaire**

**Nut Allergy Questionnaire**

This questionnaire asks you about your nut allergy. The word ‘nuts’ is used throughout this questionnaire to refer to any kind of nuts e.g. Brazil nut, Walnut, Almond, Cashew, etc.) including Peanuts. This study is being run in collaboration with doctors and nurses from the Allergy Clinic at Southampton. If you have any questions, or need help completing this questionnaire, please contact the clinic on 02380 796160.

**Section 1 – Your allergies**

| **1. Do you have a nut allergy?** |  | Yes |  | No |
| --- | --- | --- | --- | --- |

**If No -** Thank you for your time, but there is no need to continue completing this questionnaire

**2. If yes – Which type/s of nuts are you allergic to?** (Please tick all that apply)

|  | Peanuts |  |
| --- | --- | --- |
|  | Brazil nut |  |
|  | Walnut |  |
|  | Almond |  |
|  | Cashew |  |
|  | Hazelnut |  |
|  | Pecan |  |
|  | Macadamia (Queensland) |  |
|  | Pistachio |  |
|  | Other nuts (Please specify which types) |  |

| **3. Are you allergic to any other foods?** |  | Yes |  | No |
| --- | --- | --- | --- | --- |

**4. If yes, which foods are you allergic to?** (Please tick all that apply)

|  | Milk |  |
| --- | --- | --- |
|  | Egg |  |
|  | Soya |  |
|  | Fish |  |
|  | Wheat |  |
|  | Shellfish |  |
|  | Sesame seeds |  |
|  | Other (Please specify) |  |

**5. In the past year have you taken routine medication for any of the following?**

(Please tick all that apply)

|  | Asthma |  |
| --- | --- | --- |
|  | Eczema |  |
|  | Hayfever |  |

**Section 2 – Your reaction to nuts**

| **6. When did you have your *first* reaction to nuts?** |  | years ago |
| --- | --- | --- |

**7. When did you *last* have a reaction to nuts?**

|  | Within the last 6 months |  |  |
| --- | --- | --- | --- |
|  | 6 months – 1 year ago |  |  |
|  | Between 1 and 2 years ago |  |  |
|  | Over 2 years (Please specify) |  | years ago |

**8. How many times have you reacted to nuts?**

(If you can’t remember exactly please indicated the approximate number)

|  | Never |  |  |
| --- | --- | --- | --- |
|  | Once |  |  |
|  | Twice |  |  |
|  | 3 times |  |  |
|  | More than 3 times |  |  |

**9. We would like to understand what it is about nuts that causes you to have a reaction. Have you ever had a reaction to:**

|  | Yes | No | Don’t know |
| --- | --- | --- | --- |

| Smelling a nut? |  |  |  |  |  |
| --- | --- | --- | --- | --- | --- |
| Touching a nut? |  |  |  |  |  |
| Eating an (invisible) trace of a nut? |  |  |  |  |  |
| Eating less than half a nut? |  |  |  |  |  |
| If yes, please specify type of nut |  |  |  |  |  |
|  |  |  |  |  |  |
| Eating more than half a nut? |  |  |  |  |  |
| If yes, please specify type of nut |  |  |  |  |  |

**Section 3 - Your worst ever reaction to nuts**

Please think about your *worst ever* reaction to nuts and answer the questions in this section based on that experience.

| **10. What food do you think caused this reaction (e.g. curry, chocolate) etc)?** | | |
| --- | --- | --- |
|  |  |  |

**11. Was this food…?**

|  | Pre-packed |  |  |
| --- | --- | --- | --- |
|  | From a restaurant |  |  |
|  | From a take away |  |  |
|  | Eaten at a party |  |  |
|  | Other (please specify) |  | |

**12. How did you react during your *worst ever* reaction to nuts?**

(Please tick all that apply)

|  | Vomiting | |  |
| --- | --- | --- | --- |
|  | Abdominal pain | |  |
|  | Rash | |  |
|  | Face swelling | |  |
|  | Tingling/ sore mouth | |  |
|  | Swelling of lips or tongue | |  |
|  | Throat tightening/ difficulty swallowing | |  |
|  | Breathing difficulties | |  |
|  | Wheeze | |  |
|  | Blue around the lips | |  |
|  | Collapse/ faint | |  |
|  | Other (please specify) |  | |

**13. How long after you were in contact with nuts did your *worst ever* reaction start?**

|  | Immediately | | ( in under 5 minutes) |  |
| --- | --- | --- | --- | --- |
| O |  |  | minutes after contact | |

**14. What treatment did you have for your *worst ever* reaction?**

(Please tick all that apply)

| None |  | Yes |  |  |
| --- | --- | --- | --- | --- |
| Antihistamine (e.g. cetirizine/ piriton syrup or tablets) |  | Yes |  | No |
| Asthma inhaler (blue- reliever) |  | Yes |  | No |
| Injectable Adrenaline (e.g. Epipen or Anapen) |  | Yes |  | No |
| Called an ambulance – paramedic assessment |  | Yes |  | No |
| and emergency treatment |  |  |  |  |

| **15. Did you go to hospital?** |  | Yes |  | No |
| --- | --- | --- | --- | --- |

**If No –** Please go to question 18

| **16. Were you admitted over night?** |  | Yes |  | No |
| --- | --- | --- | --- | --- |

**If No –** Please go to question 18

**17. If yes, were you admitted to…?**

|  | Intensive care |  |  |
| --- | --- | --- | --- |
| O | Ordinary Ward |  |  |

**18. Where were you when you had your *worst ever* reaction?**

|  | At home | |  |
| --- | --- | --- | --- |
|  | At work | |  |
|  | At school | |  |
|  | At a friend’s house | |  |
|  | At a restaurant/ café/ bar | |  |
|  | At a party | |  |
|  | Other (please specify) |  | |

**Section 4 – Your allergy care**

**19. We would like to know which places. people or sources you have used to find out about your nut allergy and the best way to manage it.**

In the boxes on the left, please mark *which* of the following information sources you have used. In the boxes on the right please indicate *how much* you used each source of information.

| Information sources  you used | Not much | A little | Quite a lot | A great deal |
| --- | --- | --- | --- | --- |

|  | GP |  |  |  |  |  |  |  | |
| --- | --- | --- | --- | --- | --- | --- | --- | --- | --- |
|  | Hospital Allergy Specialist |  |  |  |  |  |  |  | |
|  | Magazines |  |  |  |  |  |  |  | |
|  | Newspapers |  |  |  |  |  |  |  | |
|  | Books |  |  |  |  |  |  |  | |
|  | Tv and Radio |  |  |  |  |  |  |  | |
|  | Friends and family |  |  |  |  |  |  |  | |
|  | Other person with nut allergy |  |  |  |  |  |  |  | |
|  | NHS direct |  |  |  |  |  |  |  | |
|  | Food Standards Agency (FSA) |  |  |  |  |  |  |  | |
|  | Websites |  |  |  |  |  |  |  | |
|  | Other (please specify) |  |  |  |  |  |  |  |  |
|  |  |  |  |  |  |  |  |  |  |
|  |  |  |  |  |  |  |  |  | |

**20. Have you ever had any of the following tests to confirm that you have a nut**

**allergy?**(Please tick all that apply)

|  | Skin tests | |  |
| --- | --- | --- | --- |
|  | Blood tests | |  |
|  | Food challenge | |  |
|  | Other (please specify) |  | |
|  | Never had a test (Please continue to question 22) | | |

**21. Who organised these tests?**

|  | GP/Family doctor | |  |
| --- | --- | --- | --- |
|  | Hospital doctor | |  |
| = | Other (please specify) |  | |

**22. Have you ever attended a hospital allergy clinic about your nut allergy?**

|  | Yes |  | No (Please continue to question 24) |
| --- | --- | --- | --- |

**23. If yes...**

| **a) Which hospital?** |  |
| --- | --- |

| **b) When were you last seen by a hospital allergy doctor?** |
| --- |

|  | Within the last 6 months |  |  |
| --- | --- | --- | --- |
|  | 6 months – 1 year ago |  |  |
|  | Between 1 and 2 years ago |  |  |
|  | Over 2 years (Please specify) |  | years ago |

**24. Have you been advised by a doctor to carry any of the following medication for your nut allergy?** (Please tick all that apply)

| Antihistamine (e.g. cetirizine/ piriton syrup or tablets) | |  | Yes |  | No |
| --- | --- | --- | --- | --- | --- |
| Asthma inhaler (blue - reliever) | |  | Yes |  | No |
| Injectable Adrenaline (e.g. Epipen or Anapen) | |  | Yes |  | No |
| Other (please specify) |  | | | | |

**Section 5 – Your food choices**

**25. Generally who shops for the food that you eat?** (Please tick all that apply)

|  | Me | |  |
| --- | --- | --- | --- |
|  | A family member or partner | |  |
|  | Someone else | |  |
|  | I/we order food on the internet |  | |

**26. On average, how often do you personally shop for food for yourself in the following places?**

|  | | Never | | | Once a  month | | | Once a  fortnight | | | Once a week | | | 2-3 times  a week | | | Daily | | |
| --- | --- | --- | --- | --- | --- | --- | --- | --- | --- | --- | --- | --- | --- | --- | --- | --- | --- | --- | --- |
|  | | Supermarket | |  |  | |  |  | |  |  | |  |  | |  |  | |  |
|  | | Local Shop | |  |  | |  |  | |  |  | |  |  | |  |  | |  |
|  | | Coffee shop/ Café | |  |  | |  |  | |  |  | |  |  | |  |  | |  |
|  | | Restaurant | |  |  | |  |  | |  |  | |  |  | |  |  | |  |
|  | | Take away | |  |  | |  |  | |  |  | |  |  | |  |  | |  |
|  | | Other (please specify) | |  |  | |  |  | |  |  | |  |  | |  |  | |  |
|  | |  | |  |  | |  |  | |  |  | |  |  |  |  |  |  |  |

**27. How often do you do food shopping at the following stores?**

(Please tick all that apply)

|  | | Never | | | Rarely | | | Sometimes | | | Often | | | Always | | |  |  |
| --- | --- | --- | --- | --- | --- | --- | --- | --- | --- | --- | --- | --- | --- | --- | --- | --- | --- | --- |
|  | | Tesco | |  |  | |  |  | |  |  | |  |  | |  |  | |
|  | | Waitrose | |  |  | |  |  | |  |  | |  |  | |  |  | |
|  | | Sainsbury’s | |  |  | |  |  | |  |  | |  |  | |  |  | |
|  | | Asda | |  |  | |  |  | |  |  | |  |  | |  |  | |
|  | | Morrison’s | |  |  | |  |  | |  |  | |  |  | |  |  | |
|  | | Lidl | |  |  | |  |  | |  |  | |  |  | |  |  | |
|  | | Aldi | |  |  | |  |  | |  |  | |  |  | |  |  | |
|  | | Marks and Spencer | |  |  | |  |  | |  |  | |  |  | |  |  | |
|  | | Co-Op | |  |  | |  |  | |  |  | |  |  | |  |  | |
|  | | Local shops | |  |  | |  |  | |  |  | |  |  | |  |  | |
|  | | Other (please specify) | |  |  | |  |  | |  |  | |  |  | |  |  | |
|  | |  | |  |  | |  |  | |  |  | |  |  |  |  |  |  |

**Section 6 – Other members of your household (who eat with you at home)**

**28. Is there anybody else, who eats with you at home, who is allergic to foods?**

|  | Yes |  | No (please continue to question 30) |
| --- | --- | --- | --- |

**29. If yes, which foods are they allergic to?** (Please tick all that apply)

|  | Peanuts | |  |
| --- | --- | --- | --- |
|  | Other nuts (please specify) |  | |
|  | Milk | |  |
|  | Egg | |  |
|  | Soya | |  |
|  | Fish | |  |
|  | Wheat | |  |
|  | Shellfish | |  |
|  | Sesame seeds | |  |
|  | Other (please specify) | |  |

**Section 7 Background information**

Finally we would like to ask you a few standard questions about yourself to help us characterise the people who have taken part in our research

| **30. Date of Birth:** | **D** | **D** | **M** | **M** | **Y** | **Y** |
| --- | --- | --- | --- | --- | --- | --- |

| **31. Gender:** | **Male** | **M** | **Female** | **F** |
| --- | --- | --- | --- | --- |

**32. Ethnicity:**

| **White** | | **Asian or Asian British** | | **Black or Black British** | |
| --- | --- | --- | --- | --- | --- |
|  | Any white background |  | Indian |  | Caribbean |
| **Mixed** | |  | Pakistani |  | African |
|  | White and Black Caribbean |  | Bangladeshi | **Chinese** | |
|  | White and Black African |  |  |  | Chinese |
|  | White and Asian |  | **Other (please specify)** | | |

**33. What is you highest educational qualification?** (Please tick one box only)

|  | Degree or degree equivalent and above |
| --- | --- |
|  | Higher Education to less than degree level (e.g. HND) |
|  | A level/ Scottish Higher/ Vocational level 3 and equivalent |
|  | O level/ GCSE/ Vocational level 2 and equivalent |
|  | No qualifications |

As mentioned in the letter enclosed with this questionnaire, we are looking for participants to take part in our research. When you have read this letter we would like to know if you would be interested in taking part in either of the research studies we are conducting. At this stage we are only asking for your permission to send you more information. You are under no obligation to take part.

|  | Yes |  | No |
| --- | --- | --- | --- |

**Would you be interested in receiving further information about Study A – The Shopping Study?**

|  | Yes |  | No |
| --- | --- | --- | --- |

**Would you be interested in receiving further information about Study B – The Questionnaire Study?**

The information about these studies will be sent to you from our research team at the University of Surrey. This will involve your contact details being passed to the research team. Please provide consent below that you are happy to be contacted by the research team.

| **Consent section**  I consent to my contact details being passed to the University of Surrey Research Team. I understand that all personal data relating to research participants is held and processed in the strictest confidence and will be destroyed on completion of the study, and in accordance with the Data Protection Act (1998). I understand that all personal data will be destroyed on completion of the study.  I understand that I will only receive further details about the research project, and that I am not at this stage consenting to take part in either of the research studies. I understand that I am under no obligation to take part in this research.  **Signed** ______________________________  **Date** _____________________________  **Name** (block capitals please)___________________________________________________  **Address**___________________________________________________________________  ___________________________________________________________________________  **___________________________________________________________________________**  **Contact phone number** ______________________________________________________  **Best time to contact**_________________________________________________________ |
| --- |
