# Supplementary material for: Beyond Labelling: What Strategies Do Nut Allergic Individuals Employ to Make Food Choices? A Qualitative Study
Source: PLoS One. 2013 Jan 29;8(1):e55293. doi: 10.1371/journal.pone.0055293 (PMC3558473; doi:10.1371/journal.pone.0055293)
Supplement: File S2 — Think aloud training & accompanied shop instructions. (DOCX) [file pone.0055293.s002.docx]

**A. Think Aloud Training**

The details of how participants were trained in the ‘think aloud’ protocol are presented in box 1. The training lasted approximately 10 minutes and was undertaken exactly before the accompanied shop.

# Box 1

As I have already mentioned, in this task I would like you to think aloud while you are doing your usual shopping. What I mean by thinking out loud is to say everything you are thinking while you are shopping. So maybe this will be what you are looking at, reasons why you are choosing to buy it, or reasons why you aren’t buying it and so on…

When I say ‘everything you are thinking’ I literally mean just that, no matter how fleeting or apparently trivial the thoughts may be. That means including everything that you are looking at and registering mentally, as well as anything you are doing.

The idea is that you continue talking as much as possible about what you are thinking, rather than you and I having a conversation, so you will find that I will follow you and observe what you are doing, but not talk a lot to you. When you are talking out loud it is fine for you to aim that at me, so you feel you are not talking to yourself!

In terms of your allergy you don’t need to specifically talk about this (for my benefit) unless it is something that you are thinking about. So if it was particularly relevant to you choosing an item and you are thinking about your allergy then do talk out loud about that, but if it is not and you are not choosing food with this in mind then just carry on as usual verbalising whatever else you are thinking.

I have a few practice examples here as it can help to get used to talking out loud. I will demonstrate the first example then if you are happy to have a go you can try the following example.

It is also really useful if you mention what in particular you are looking at, for the benefit of the tape recording.

So I’ll start now with an example of how I might think aloud if I was trying to buy a toaster out of a catalogue.

“I am looking at the Cookworks toaster, it’s 14.69 – so it seems nice and cheap and within my budget. It toasts 4 slices of bread which is handy. But then there’s this Breville one which is 16.99, a bit more expensive and only toasts 2 slices of bread. Although I prefer the look of this one as it is silver and black and I don’t really need the toaster to toast 4 slices of bread, so I think I’ll choose this one.”

Could you now do the same as though you wanted to buy a kettle and you were choosing between these two?

Would you like to have a go at talking out loud about these irons too?

**B. Instructions for accompanied shop**

The exact instructions that were provided to participants for the accompanied shop task are presented in box 2.

# Box 2

- Carry out your shopping in your usual way. Don’t rush on my account. It may feel strange to have me following you around.

- Think aloud at all times

- I will prompt you if you fall silent for more than 10 seconds and I will probably say: ‘Keep thinking aloud’; or ‘What are you thinking’; or ‘What are you looking at?’ I will not embark on any conversation with you.

- I may make some notes during the shop, don’t worry about this

- Just to reassure you, I’m not here to judge what you actually buy.
